# The Atlanta Society of Medicine

**Published:** 1888-07

**Authors:** 


### THE ATLANTA SOCIETY OF MEDICINE.

---

THE annual banquet of the Atlanta Society of Medicine, which occurred at the Markham House June 1st, 1888, at 9 o'clock p. m., was the most enjoyable affair of the kind that has ever taken place in this city.

Besides the thirty-five members of the Atlanta Society of Medicine, there were present upon this occasion representatives of the press and legal profession. They were ushered into the dining room a few minutes past 9 o'clock and invited to partake of the most elegant and sumptuous repast that it has ever been our pleasure to behold. The table was beautifully decorated with flowers and the *menu* served in a perfect manner.

Dr. W. S. Elkin, chairman of the Committee of Arrangements, introduced, as master of ceremonies for the evening, Dr. J. C. Olmsted, who announced the toasts and called for responses in a pleasing and dignified style that to him alone is characteristic. The following is a list of the toasts:

OUR STATE—GOV. JNO. B. GORDON.

Georgia! The Empire State of the South. May the light of her past virtues ever illumine her future progress.

THE PRESS—EVAN P. HOWELL.

"Through the rare felicity of the times you are permitted to think what you please and to publish what you please."

THE LAWYERS—J. CARROLL PAYNE.

"Piecemeal they win this acre first, then that;
Glean on, and gather up the whole estate."

OUR GUESTS—DR. ROBERT BATTEY.

"If they had been forgotten, it had been as a gap in our feast."

THE ATLANTA SOCIETY OF MEDICINE—DR. V. O. HARDON.

Let her past success be an earnest of her future usefulness.

WOMAN—DR. J. B. BAIRD.

"O woman! lovely woman! Nature made thee to temper man; we had been brutes without you. Angels are painted fair to look like you."

OUR COLLEGES—DR. W. P. NICOLSON.

"Two souls with but a single thought,
Two hearts that beat as one."

THE PHYSICIANS—DR. J. S. TODD.

"How merry are we,
Though doctors we be;
We humbug the public
And pocket the fee."

The responses to the sentiments of "Our State" and "Our Guests" were not responded to owing to the absence of Gov. Gordon and Dr. Battey. The former was unable to attend on account of sickness, and the latter telegraphed at a late hour that owing to the illness of a patient he was compelled to forego the pleasure of attending the banquet, much to his disappointment and regret. Happy and bright speeches were made in response to the other sentiments, after which calls for speeches were responded to by Drs. Kendrick, Olmsted, Westmoreland, Earnest, Cooper, Elkin and many others. One o'clock a. m. warned the master of ceremonies that a motion for adjournment was in order, but this was not made until a unanimous vote of thanks was tendered Mr. Maxwell, proprietor of the Markham House, for the elegant banquet, which had afforded so much pleasure to all present.

### ITEMS.

THE AMERICAN RHINOLOGICAL ASSOCIATION will hold its Sixth Annual Meeting at Cincinnati, Ohio, September 12, 13 and 14, 1888.

THE SOUTHERN SURGICAL AND GYNECOLOGICAL SOCIETY will meet in Birmingham, Ala., September 10th, 1888. Dr. W. E. B. Davis is the Secretary.

A TIMELY article in the July *Century* is "Disease Germs and How to Combat Them." It will be accompanied by a frontispiece portrait of Pasteur, who has made disinfection and fermentation a longer study than hydrophobia, although it is with the latter that his name is more intimately associated with the public mind.

ROBERT LOUIS STEVENSON, in his paper on "Popular Authors," which will be published in the July *Scribner's*, says: "Once I took the literary author at his own esteem; I behold him now like one of those gentlemen who read their own MS. descriptive poetry aloud to wife and babes around the evening hearth, addressing a mere parlor coterie and quite unknown to the great world outside the villa windows. At such pigmy reputation, Reynolds, or Cobb, or Mrs. Southworth can afford to smile. By spontaneous public vote, at a cry from the unorganic masses, these great ones of the dust were laureled."

PROGRAMME OF THE ANNUAL MEETING OF THE AMERICAN ASSOCIATION OF OBSTETRICIANS AND GYNECOLOGISTS, TO BE HELD IN WASHINGTON, D. C., SEPTEMBER 18, 19 AND 20, 1888.—The President's annual address, William H. Taylor, Cincinnati. *Discussion*—EXTRAUTERINE PREGNANCY.—1. Pathology. 2. Diagnosis. 3. Treatment. [a] Medical. [b] Electrolytic. [c] Surgical. The relations of the Abdominal Surgeon to the Obstetrician and Gynecologist, Albert Vander Veer, Albany. Operation for an unusual case of subserous uterine fibroid, Hampton Eugene

Hill, Saco, Me. Drainage in abdominal and pelvic surgery, Joseph Price, Philadelphia. Double ovariotomy during pregnancy ; a successful case going on to full term, William Warren Potter, Buffalo. The indications for artificial aid in labor, Thomas Opie, Baltimore. The technique of Vaginal Hysterectomy, James H. Etheridge, Chicago. The surgical treatment of the perineum, William H. Wathem, Louisville. Laparotomy in peritonitis, E. E. Montgomery, Philadelphia. Tumors of the abdominal wall, Charles A. L. Reed, Cincinnati. Uterine fibroids ; their diagnosis and treatment, Thomas J. Maxwell, Keokuk. Desmoid (fibroid) tumors of the abdominal wall, Edward J. Ill, Newark. Ruptured perineum, J. Henry Carstens, Detroit. A contribution to the study of pelvic abscess, Clinton Cushing, San Francisco. The female perineum; its anatomy; physiological function, and methods of restoration after injury. This paper will be illustrated with lime-light and screen, Henry O. Marcy, Boston. Heart failure in the puerperium, Thomas Lothrop, Buffalo. Treatment of suppurative peritonitis, William H. Myers, Fort Wayne. Operative treatment in uterine carcinoma, George R. Shepard, Hartford. The reflexes reflected ; or some things that retard progress in gynecic surgery, Joseph Eastman, Indianapolis. Some points in relation to the diagnosis of pregnancy in the early months, James P. Boyd, Albany. Vaginal tamponnement in the treatment of prolapsed ovaries, W. P. Manton, Detroit. Mr. Lawson Tait, F. R. C. S. E., Birmingham, England, will also present a paper on "The methods of success in abdominal surgery." *Note.*—Mr. Lawson Tait, Dr. Franklin Townsend, Dr. E. E. Montgomery, Dr. Charles A. L. Reed, Dr. A. Vander Veer and others will participate in the discussion on Extrauterine Pregnancy. The full announcement of the topics that each referee will speak to will be made in the final programme to be issued in August. William H. Taylor, M. D., President ; William W. Potter, M. D., Secretary.

